# Editorial: Addressing insulin resistance and hyperinsulinemia for cardiovascular disease prevention

**DOI:** 10.3389/fcvm.2026.1802226

**Published:** 2026-03-16

**Authors:** Serafino Fazio, Giacomo Pucci, Loredana Tibullo, Valeria Fazio, Valentina Mercurio

**Affiliations:** 1 Federico II University Hospital, Naples, Italy; 2 Universita Degli Studi di Perugia - Polo Scientifico Didattico di Terni, Terni, Italy; 3 Azienda Ospedaliera di Caserta Sant Anna e San Sebastiano, Caserta, Italy; 4 U.O.C. Cardiologia, Presidio Ospedaliero Santa Maria Delle Grazie, Napoli, Italy

**Keywords:** cardiovascular disease, hyperinsulinemia, insulin resistance, prevention, type 2 diabetes

Despite the significant progress made in the prevention and treatment of cardiovascular diseases over the last 3 decades, they remain the leading cause of death (1). Furthermore, due to the aging population and the advent of interventional therapies (coronary angioplasty and antiarrhythmic device implantation), which allow us to overcome most episodes of acute myocardial ischemia, the incidence of chronic heart failure (HF) is progressively increasing, both with reduced ejection fraction (HFrEF) and preserved EF (HFpEF). HF has a prevalence of approximately 2% in the general population, but it increases to over 10% in the population > 65 years of age and is the most common cause of hospitalizations for this demographic, causing enormous health expenditure in Western countries (2). It would seem that we may have missed something in the management and prevention of cardiovascular disease. In reality, for years, medicine has been addressing diabetes as a pathology with a serious negative impact on the cardiovascular system, and physicians treat diabetic patients with a focus on strict prevention, according to guidelines, as if they have already developed a cardiovascular condition (3). In fact, if we examine in more depth a patient with recent-onset type 2 diabetes, evident cardiovascular damage is already present. Type 2 diabetes is known to be preceded by many years (even up to 15) of insulin resistance (IR), which is its main cause (4, 5). IR is characterized by constant chronic hyperinsulinemia (increased circulating levels of insulin, HI), as the pancreas secretes greater amounts of insulin in order to maintain blood sugar levels within a normal range (4, 5). However, there is a great deal of scientific literature that shows how the increased circulating levels of insulin can be very dangerous to the cardiovascular system (6, 7). In fact, it has been shown that chronically raised circulating levels of insulin associated with IR largely alter vascular homeostasis towards vasoconstriction by increasing endothelin-1 (ET-1) secretion while reducing nitric oxide (NO) availability and increasing norepinephrine secretion (6, 8, 9), therefore causing endothelial dysfunction and increasing the possibility of developing hypertension. Furthermore, there are other mechanisms by which HI may stimulate hypertension. By activating its tubular receptors in the kidney, insulin increases sodium and water reabsorption, expanding blood volume (10). Furthermore, insulin and the renin-angiotensin-aldosterone system (RAAS) are in a mutual regulatory relationship. An overactive RAAS, especially with high levels of Angiotensin II, promotes insulin resistance, inflammation, and vasoconstriction, contributing to diabetes and hypertension. Conversely, insulin stimulates RAAS, regulating enzymes and receptors (11).

Insulin is also a growth factor; by binding to insulin-like growth factor receptors, it stimulates the growth of endothelial and vascular smooth muscle cells and promotes the proliferation of renal mesangial cells. In addition, HI, both directly and indirectly, when causing hypertension, produces enhancement of left ventricular mass, with increased stiffness and diastolic dysfunction (12–14), consequently facilitating the development of HFpEF. We decided to write this editorial, “Addressing insulin resistance and hyperinsulinemia for cardiovascular disease prevention,” to stimulate the scientific community to address a cardiovascular risk factor that has been neglected for too long, namely, IR associated with HI. This condition is very prevalent in the general population in developed and developing countries (with an average of 30%, reaching 50% in particular areas) and is progressively increasing (15). This is mainly due to the progressive change in our lifestyle, generally characterized by an increase in caloric intake, including excessive sugar consumption, and a reduction in physical activity. It is very likely that by intervening promptly on IR with screening and treatment, we will be able to reduce the number of cases of type 2 diabetes and prevent cardiovascular damage resulting from HI. The manuscripts published in this special issue are interesting, and all show that surrogate markers of insulin resistance, including the most widely used TyG index, are effective markers not only of metabolic risk (such as for example of hyperuricemia) Zhou et al., Liu et al., Shi et al., Wu et al. and inflammatory risk He et al., Wu et al. but also of cardiovascular risk He et al., Huang et al., Cui et al., Chen et al., Wang et al., Zhu et al. and, in some studies, of increased mortality risk Chen et al., Li et al. Furthermore, two studies also showed that, in some particular patients (in maintenance hemodialysis), TyG was associated with a significantly increased risk of heart failure and, in patients with chronic heart failure, with an increased risk of hospital readmission for heart failure Zhang et al., Li et al. One large study (6,539 subjects) used the estimated glucose disposal rate (eGDR), a well-known index of IR, with lower values indicating greater insulin resistance, to verify the link between eGDR and the occurrence of cardiovascular disease (CVD) in individuals exhibiting Cardiovascular-Kidney-Metabolic (CKM) syndrome stages 0–3. It was conducted on Chinese individuals from the CHARLS study and had the onset of cardiovascular events as its primary outcome. During follow-up, 1,656 (26%) events occurred. The study concluded that, in a population with Cardiovascular-Kidney-Metabolic (CKM) syndrome stages 0–3, there is a non-linear relationship between eGDR and CVD risk, suggesting that an IR index such as eGDR can be considered for cardiovascular risk assessment in this type of patient Tan et al.

All studies published in this special issue strongly support the idea that IR/HI increases cardiovascular risk and that its presence should therefore be taken into account in various types of patients.

This demonstrates that we can no longer delay in considering IR/HI as an important and independent risk factor for cardiovascular disease, which scientific societies and health institutions will need to take into account in screening campaigns Fazio et al.

**Figure 1:**
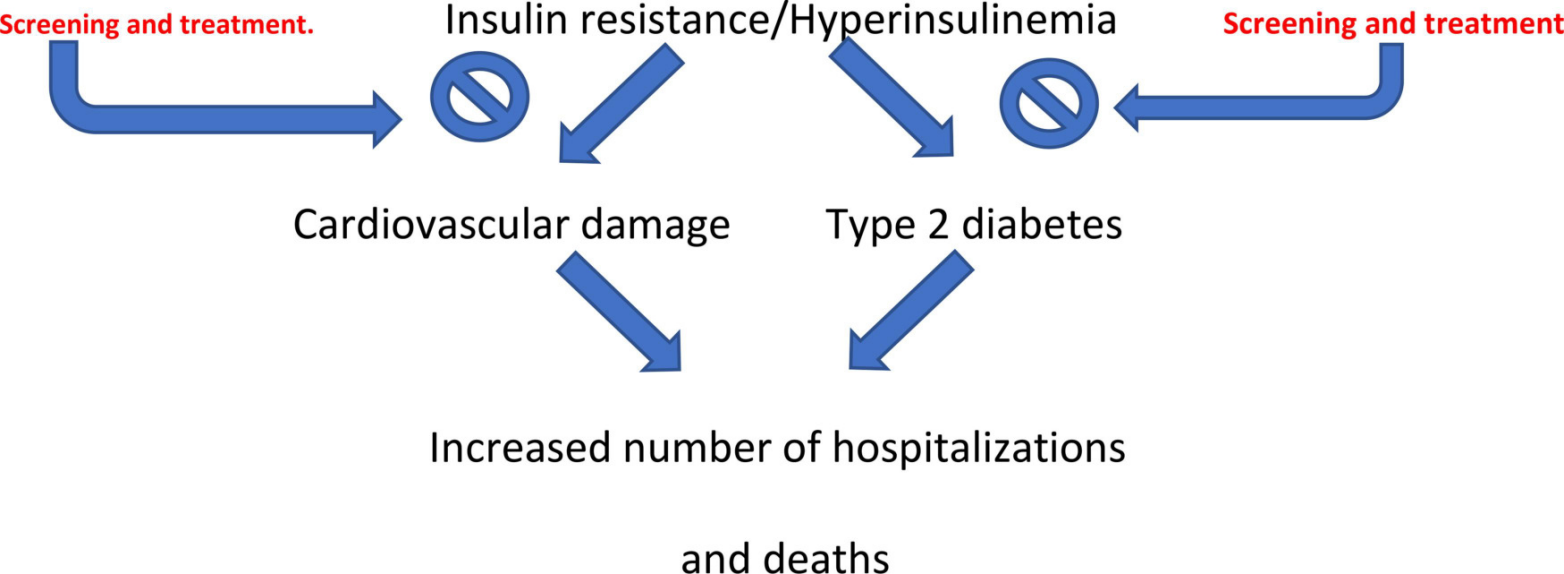
Early intervention through screening and intervention on IR/HI could significantly inhibit the progression of this condition towards the development of cardiovascular disease and type 2 diabetes, resulting in a reduction in the number of hospitalizations and deaths.

It is likely that by identifying and treating IR promptly in the general population, we will be able to reduce the continuously growing prevalence of type 2 diabetes and the progression of this condition towards cardiovascular complications. Over time, this could lead to a reduction in hospitalizations and mortality, with significant social and health benefits (Figure 1).
